# Optimizing the Radiopacity of an Injectable Polymer on Fluoroscopy used for Treatment of Type II Endoleak After Endovascular Aneurysm Repair

**DOI:** 10.1007/s13239-025-00779-w

**Published:** 2025-03-24

**Authors:** Jeffrey R. Nagel, Erik Groot Jebbink, Stefan P. M. Smorenburg, Arjan W. J. Hoksbergen, Rutger J. Lely, Michel Versluis, Michel M. P. J. Reijnen

**Affiliations:** 1 Multi-Modality Medical Imaging group, University of Twente, Enschede, The Netherlands; 2 Physics of Fluids group, University of Twente, Enschede, The Netherlands; 3 Department of Surgery, Rijnstate, Arnhem, The Netherlands; 4 Department of Surgery, Amsterdam UMC location Vrije Universiteit, Amsterdam, The Netherlands; 5 Department of Radiology, Amsterdam UMC location Vrije Universiteit, Amsterdam, The Netherlands

**Keywords:** Abdominal aortic aneurysm, Endoleak, Injectable embolic agent, Radiopacity, Tantalum

## Abstract

**Purpose:**

Type II endoleaks (T2EL) are a common complication after endovascular aneurysm repair. AneuFix is a newly designed elastic polymer for T2EL. AneuFix contains tantalum for visualization during fluoroscopy, which is crucial for monitoring the polymer in the side branches. The purpose of this study was to find the lowest concentration tantalum that is sufficient for safe injection in the aneurysmal sac.

**Methods:**

AneuFix polymer with tantalum concentrations between 0 and 30% was injected into endoleak phantoms, connected to a pulsatile flow setup and with a realistic background for fluoroscopy. Furthermore, the radiopacity was investigated on fluoroscopic systems from three different vendors, using static phantoms. Results from both the dynamic and static phantoms were qualitatively evaluated by 10 clinical experts.

**Results:**

Concentrations of ≥ 20% tantalum were consistently detected within the first 5 mm after entering the side branch, with a corresponding contrast-to-noise ratio of 2.23 ± 0.21. Furthermore, sufficient detectability scores (of at least 3 out of 5) were given to ≥ 15% tantalum. Significant differences were found in detectability scores on different fluoroscopic systems, using the default lowest-radiation-dose scan protocol for each system.

**Conclusions:**

This study showed that tantalum concentrations ≥ 20% are consistently detected on fluoroscopy in the specified region. Compared to the original 30%, this would reduce imaging artifacts from high attenuation and scattering on follow-up imaging, while retaining sufficient detectability during injection. However, because of differences in fluoroscopic systems and scan protocols between hospitals, the combination of tantalum concentration and scan protocol should be optimized for each clinical setting.

**Supplementary Information:**

The online version contains supplementary material available at 10.1007/s13239-025-00779-w.

## Introduction

Abdominal aortic aneurysm (AAA) is a localized dilatation of the abdominal aorta, resulting from a weakened vessel wall. The predominant treatment for patients with an AAA with a suitable anatomy is endovascular aneurysm repair (EVAR), where a stent graft is placed in the aorta to guide the blood flow and thus relieve pressure from the weakened vessel wall. While EVAR is associated with better short-term outcomes compared to open surgery [1], lifelong imaging surveillance is recommended due to the occurrence of late complications, most importantly endoleaks. The most common type of endoleak is type II (T2EL), resulting from retrograde blood flow through lumbar arteries or the inferior mesenteric artery (IMA) with an incidence of 20-40%, and up to 10% of these T2EL persist at 2 years. The presence of persistent T2EL may result in inferior aneurysm sac regression rates, and there is growing evidence that sac regression at 12 months after EVAR is a predictor for better outcomes with regard to reinterventions, complications and survival [2–4].

According to the most recent European guidelines an intervention for T2EL is only indicated in case of significant AAA growth (≥1 cm) [5]. The main treatment method for T2EL is embolization of the supplying arteries and/or the endoleak cavity, either transarterial or via translumbar puncture of the aneurysm sac, both with mixed technical and clinical success rates [6, 7]. During the last decade there has been a growing interest in injectable embolic agents for treatment of T2EL. Their main advantages are in situ solidification and the ability to embolize smaller peripheral vasculature, which can also be adjusted by the composition of the material. An elastic polymer that was specifically designed for the treatment and prevention of T2EL is AneuFix (TripleMed, Geleen, The Netherlands). A pivotal clinical trial is currently ongoing to obtain CE certification [8]. This polymer was developed for injection under fluoroscopic guidance and contains 30% tantalum (Ta) for radiopacity. Tantalum is a radiopaque metal that is widely used for a variety of medical applications, from contrast agents to implants.

Sufficient radiopacity of the embolic agent is paramount for good detectability, to monitor and avoid too deep penetration in aortic side branches, which could result in spinal cord ischemia and/or bowel ischemia. AneuFix was initially designed with 30% Ta, to ensure good detectability. However, a high percentage of Ta in the aneurysmal sac will create high attenuation and scattering artifacts on follow-up imaging such as computed tomography (CT), hampering the clinical assessment of the treatment effectiveness as endoleaks can no longer be reliably assessed. Therefore, balancing the Ta concentration in the polymer is crucial: high enough for sufficient detectability during injection, and as low as reasonably possible to minimize scatter artifacts during follow-up imaging.

The objective of this study was to investigate the minimally required Ta concentration in the AneuFix polymer, for sufficient detectability during injection under fluoroscopy. Since detectability may differ per fluoroscopic system, the systems of three manufacturers were also compared. This was achieved by in vitro measurements using static and dynamic endoleak phantoms and evaluation by clinical experts combined with analysis of the contrast-to-noise ratio (CNR).

## Materials and Methods

### Polymer Injection in T2EL Mimicking Phantoms

**Fig. 1:**
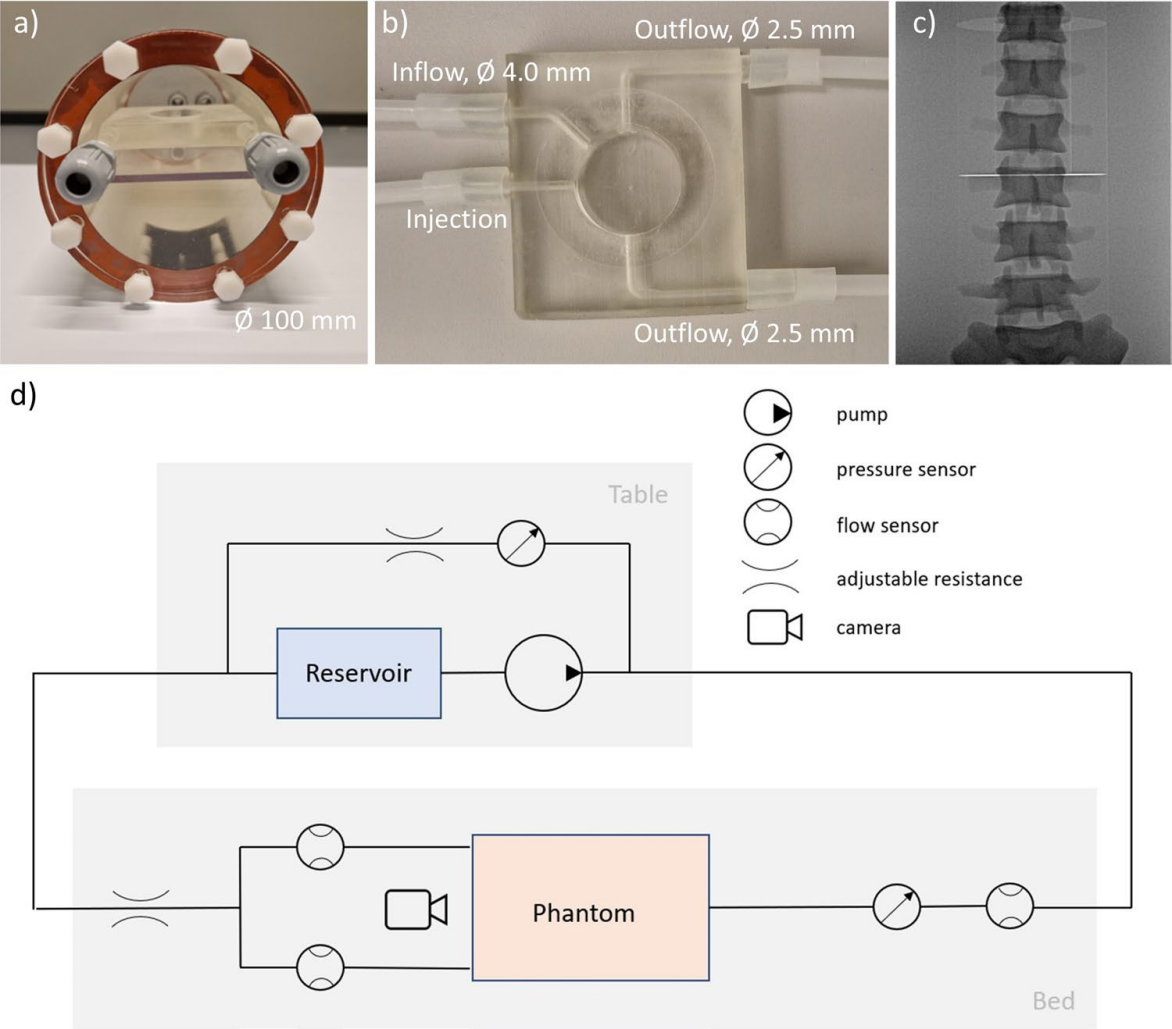
**a** Assembled endoleak phantom with the insert placed inside the cylindrical container (outer diameter 100 mm), **b** insert with in- and outflow diameters, **c** fluoroscopy image of abdomen phantom, showing the 3D printed spine, **d** schematic image of flow setup

Phantoms simulating a T2EL were designed and fabricated. The phantoms consisted of a reusable cylindrical casing (Fig. 1a) and disposable 3D printed 40 mL endoleak inserts (Fig. 1b) that were placed in the centre of the cylinder. The material used for the casing and the inserts was Clear Resin V4 (Formlabs, Somerville (MA), USA). The volume of the cavity in the inserts was based on the typical T2EL volume in an ongoing clinical trial (NCT04307992). The inserts contained a centred injection channel, a 4 mm diameter inflow channel and two 2.5 mm side branches, simulating the typical dimensions of the IMA and lumbar arteries, respectively [9].

The endoleak phantom was placed inside a customized phantom of the abdomen (QRM GmbH, Möhrendorf, Germany), featuring tissue-mimicking material (Hounsfield unit (HU) = 35), a spine (HU = 700), kidneys, part of the liver and spleen, to provide a realistic background (Fig. 1c) during fluoroscopy and CT.

The phantom was connected to a flow circuit (Fig. 1d), with a peristaltic pump (L/S Easy-Load II, Masterflex SE, Gelsenkirchen, Germany) to provide pulsatile flow. The flow rate was set to 90 mL/min, simulating a mean peak systolic velocity of 15.8 cm/s in the lumbar arteries [10], and the pressure was set to ~ 120 mmHg with clamp-on resistances. The water reservoir was heated to 37°C, as the polymer was designed for use at body temperature. Flow sensors (Sonoflow CO.55/060 V2.0, Sonotec, Halle, Germany) and pressure sensors (Manometer, Welch Allyn, Inc., Skaneateles Falls, NY, USA) were included to monitor local flow and pressure.

AneuFix polymer currently has a concentration of 30% Ta [8]. To find the optimal Ta concentration a range of lower concentrations was investigated – 0% (as a control), 2.5%, 5%, 7.5%, 10%, 12.5%, 15%, 20%, 25% and 30% Ta. For each of these concentrations, a specific batch of AneuFix was produced with the required concentration Tantalum and the same properties otherwise. Polymer was injected into the endoleak phantoms through a 6 French sheath (Engage Hemostasis Introducer, Abbott, Lake Bluff (IL), USA). For each concentration, three phantoms were made and filled. To ensure repeatibility, polymer injection was performed using a customized machine-controlled injection pump, set to a continuous 40 mL/min injection rate, filling the endoleak phantom in one minute. The polymer injection was recorded by a camera at the back side of the phantom bore hole, providing the exact polymer location.

Fluoroscopy videos during injection were obtained with a Siemens Artis Pheno hybrid OR system using a default clinical scan protocol for the abdominal region (FL (-) Angio (flavor2) protocol, 90 kV, 240 mA, 5 fps). The detector and phantom positions were stationary between measurements.

### Multi-Vendor Fluoroscopy on Static Phantoms

**Fig. 2:**
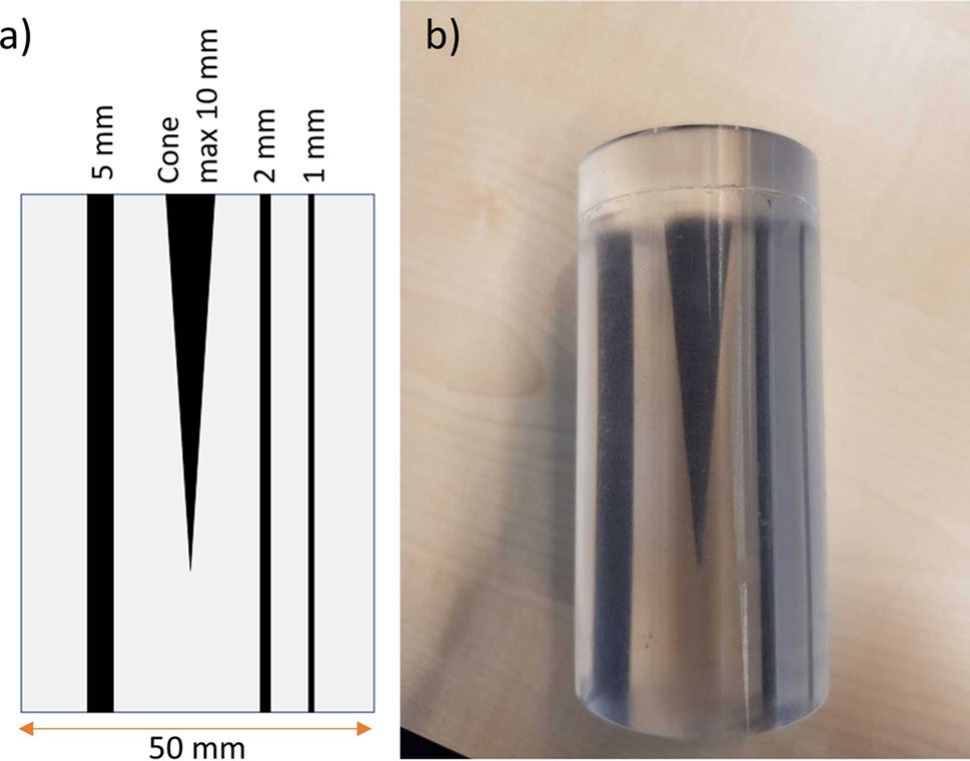
Phantoms for multi-vendor study; **a** schematic design, showing the channels with different diameters and the cone, **b** picture of one of the phantoms

The second evaluation of the Ta concentration was a multi-vendor fluoroscopy analysis. Static, prefilled phantoms were fabricated for a comparison of polymer detectability on different vendors of fluoroscopic systems. These phantoms consisted of a polydimethylsiloxane (PDMS) cylinder (Sylgard 184, Dow Corning, Midland (MI), USA) and included channels with diameters of 1 mm, 2 mm and 5 mm and a cone (Fig. 2a and b). Polymer with a Ta range identical to the endoleak phantoms was injected in the channels and cones of ten phantoms, one for each concentration. The cylinders were placed inside the aforementioned customized abdomen phantom.

Fluoroscopy imaging was obtained for each Ta concentration with three different X-ray hybrid operating room systems; an Artis Pheno (Siemens, Erlange, Germany), an Azurion (Philips, Amsterdam, The Netherlands) and a Discovery (General Electric Healthcare, Chicago (IL), USA). For each of these fluoroscopy systems the lowest-dose protocol was chosen for the abdominal region (Table 1). The phantoms were centred under the detector plate and the detector distance was set to 105 cm.

**Table 1:** Default lowest-dose scan protocols for three fluoroscopic systems, and typical values for the voltage (kV), current (mA) and product of current and exposure time (mAs)

Fluoroscopic system	Protocol	Typical kV	Typical mA	Typical mAs
Siemens Artis Pheno	FL (-) Angio (flavor2)	90	240	75
Philips Azurion	Abdomen low	85	10	5
GE discovery	Abdomen dose limited	75	100	15

### Evaluation by Clinical Experts

The obtained fluoroscopy images, for both the endoleak phantoms and static phantoms, were evaluated by clinical experts according to a predefined protocol (Supplementary Information S1). Seven interventional radiologists and three vascular surgeons, selected from four different hospitals, participated in the evaluation.

For the polymer injection, the exact moment that the polymer entered a side branch was determined by the experts, which was compared to the exact time of the polymer entering the side branches obtained from the video recordings (the ground truth). The experts were blinded to the Ta concentration. To avoid predictability bias after the first several evaluations, the starting times of the videos were randomized and half of the videos were mirrored. Each concentration was evaluated at least once by each expert, but duplicate concentrations were included as well for intra-observer variability analysis.

**Fig. 3:**
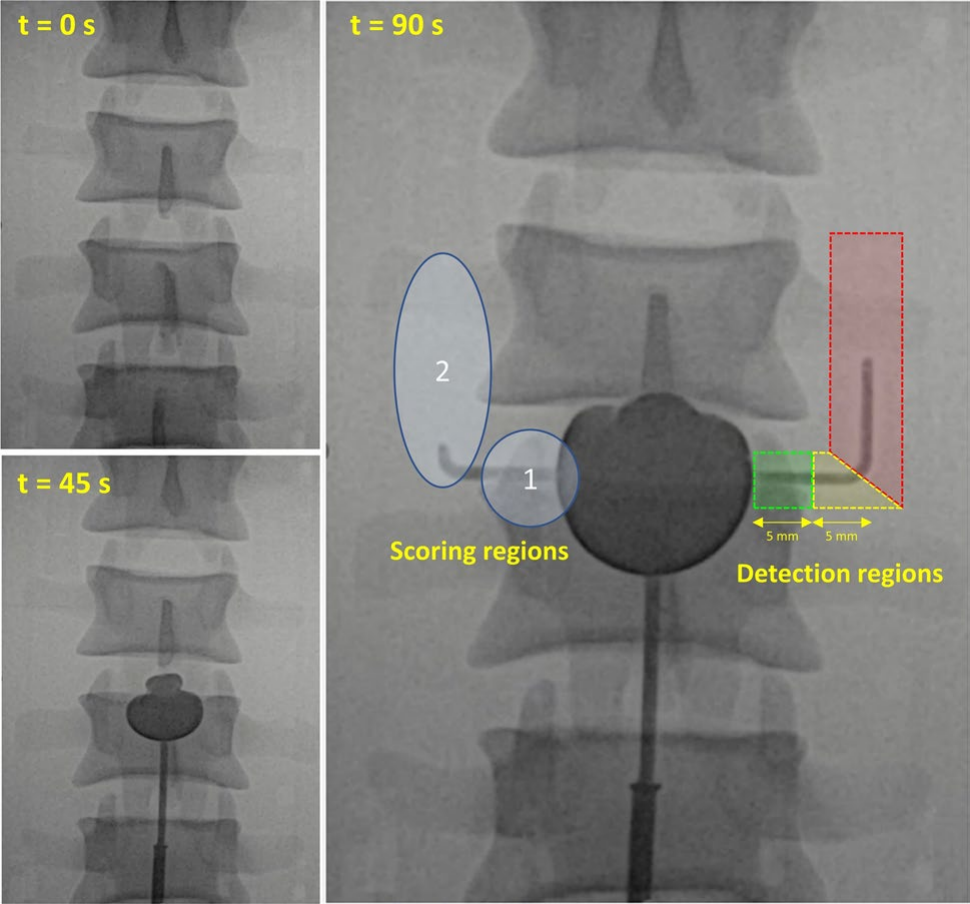
Polymer injection in the endoleak phantom at several time steps, with the first two images on the left showing the partially filled phantom and the final image on the right showing the fully filled phantom, including the classification for the detection regions; green = within 5 mm of the channel origin, yellow = between 5 and 10 mm, red = more than 10 mm. Also showing the regions for detectability scoring; region 1 is close to side branch entrance with the vertebral column in the background, region 2 further down the side branch without the vertebral column

The detection of the polymer was categorized in three regions (Fig. 3); within 5 mm of entering the side branch (green region), between 5 – 10 mm (yellow region) and more than 10 mm (red region). Safe detection was defined as a detection within 5 mm after the polymer enters the side branch. Ta concentrations for which the polymer was detected in the green region were considered sufficiently detectable. Results were normalized to the time at which the polymer reached the 10 mm point (the corner in the side branch).

The experts were also asked to score the images, both for the endoleak phantoms and for the static phantoms, on a scale of 1-5 in terms of detectability (1 = very poor, 5 = excellent).

### Contrast-to-Noise Ratio

For all fluoroscopy images, the CNR was calculated as follows:

$$CNR=\frac{{S}_{A}-{S}_{B}}{\sigma }$$

Here S_A_ and S_B_ are the mean pixel intensities of the signal region of interest and its neighbouring region (i.e. the background), respectively, and σ is the noise, typically measured as the standard deviation of the pixel intensity in the background. The mean pixel intensity was calculated for the disc-shaped cavity in the phantom, when empty and when filled with polymer, along with their respective standard deviations. The window level and window width were kept constant. The concentration Ta for sufficient detectability, determined from analysis of the clinical expert evaluation data, was then matched with the corresponding CNR.

### Statistics

The interobserver difference, defined as the average absolute difference between readings from different observers [11], was determined for the detectability scores of the clinical experts. Each fluoroscopy video of the endoleak phantoms was scored 4-5 times and with three phantoms per concentration Ta, each concentration was thus scored 12-15 times. Each fluoroscopy image of the static phantoms was scored 9-11 times. The intra-observer disagreement, defined as the average absolute difference between readings from the same observer, was assessed for each expert from evaluations of duplicate measurements. These were either duplicates of the same video/image, or duplicates of the same video/image, but mirrored.

## Results

### Detectability During Injection

**Fig. 4:**
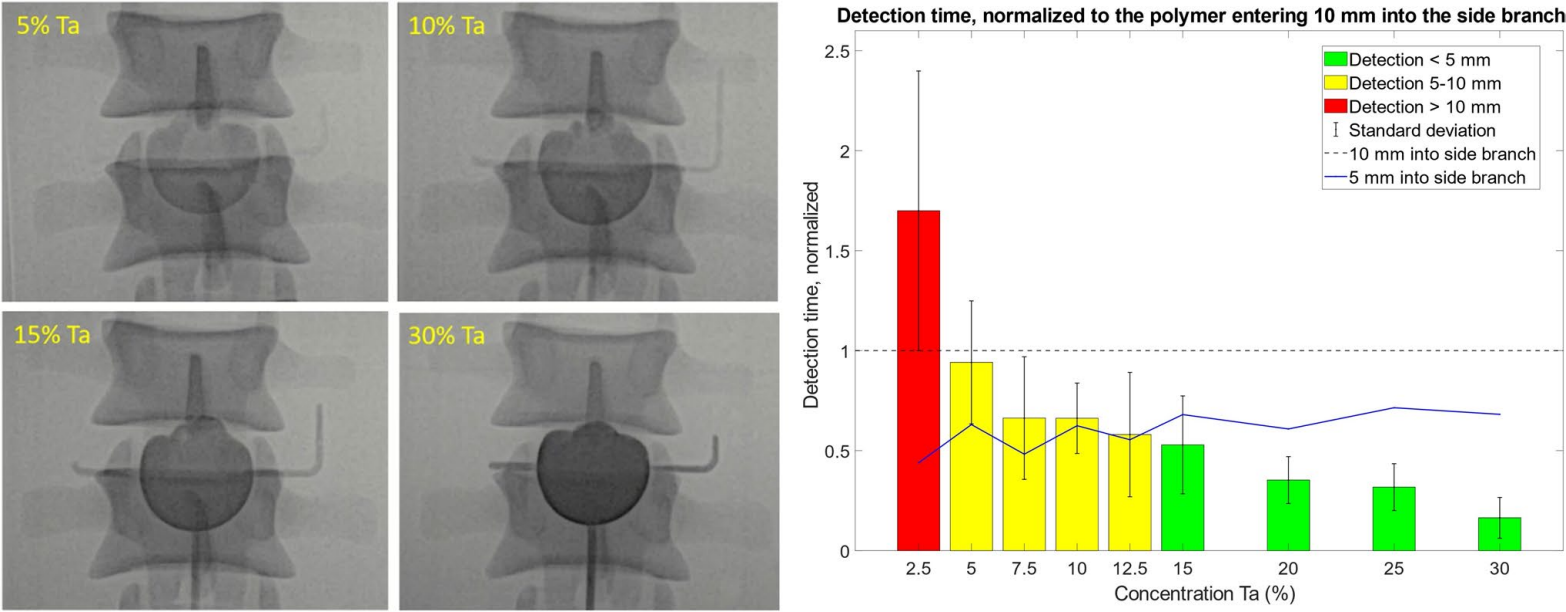
**a** Fluoroscopy images of four tantalum (Ta) concentrations; **b** Normalized detection time of the polymer, as evaluated by clinical experts, normalized to time at which the polymer entered 10 mm into the side branches (dotted line). The blue line is the moment the polymer reached 5 mm into the side branch, determined from the ground truth camera recordings

Fig. 4a shows the fluoroscopy results for four different Ta concentrations (see also Supplementary Information S2 for a video), in which a clear difference in detectability can be observed between the various concentrations. The evaluation of one clinical expert was excluded, because the monitor brightness was accidentally adjusted to 65% instead of 100%, which negatively impacted the detectability of the polymer and prevented direct comparison with evaluations of the other experts. This left the data of 9 observers for analysis of the polymer detection (Fig. 4b). Concentrations of ≥ 20% Ta were always detected within the first 5 mm after entering the side branch consistently, whereas 15% Ta was detected within the first 5 mm on average. Concentrations of 5-12.5% Ta were detected 5-10 mm from the orifice of the side branch, 2.5% Ta was only detected after 10 mm and 0% Ta was not detected at all. The slowest mean detection time was 12.0 ± 3.0 seconds for 2.5% Ta and the fastest mean detection time was 1.7 ± 1.6 seconds for 30% Ta. Lower Ta concentrations generally showed a larger standard deviation.

In addition to the detection time of the polymer entering the side branch, the experts also scored the detectability of the polymer in two regions (blue circles marked in Fig. 3). The results in Table 2 show that the detectability was scored significantly lower close to the side branch entrance, which was attributed to the presence of the vertebral column in the background of the recording.

**Table 2:**
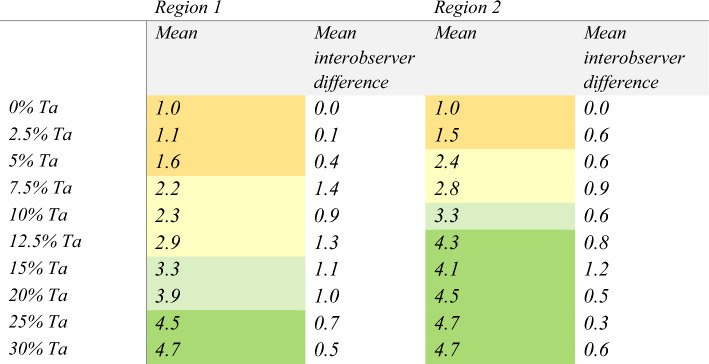
Average scores, from 1 to 5 (1 = very poor, 5 = excellent), and the mean interobserver difference of the polymer detectability near the channel entrance (region 1) and further down the side branch (region 2), evaluated by clinical experts

The scores are color-coded (<2: orange, 2 – 2.9: yellow, 3 – 3.9: light green, ≥ 4: dark green).

**Fig. 5:**
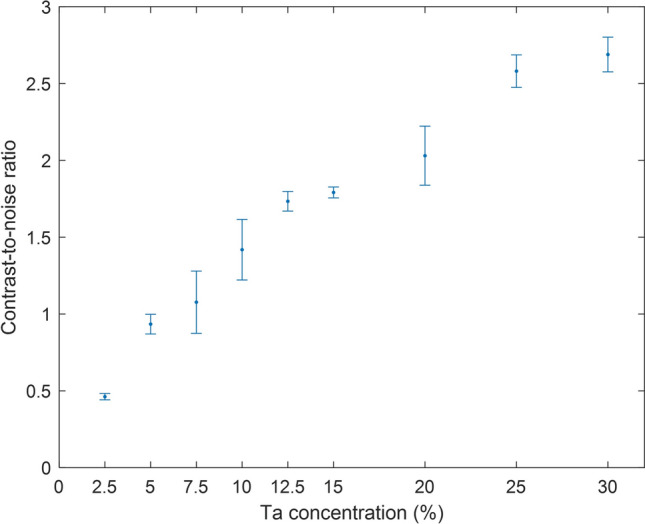
Contrast-to-noise ratio for the fluoroscopy measurements on endoleak phantoms with different tantalum (Ta) concentrations. The error bars show the standard deviation of repeated measurements (same concentration, duplicate phantoms)

The CNR of the endoleak phantom measurements for each concentration is shown in Fig. 5. Sufficient detectability scores (i.e. a score of at least 3 out of 5) were given for concentrations of ≥ 15% Ta. The intra-observer difference is 0.5.

The mean interobserver difference for the detectability scores per concentration is shown in Table 2. In region 1 the difference was highest for the intermediate Ta concentrations. A better agreement in scoring the low and high concentrations was observed, where the detectability was respectively insufficient and good. The variability in region 2 was overall lower, but no significant trend was observed. The intra-observer difference was 0.5.

### Multi-Vendor Fluoroscopy Comparison

The detectability scores, on a scale of 1 (very poor) to 5 (excellent), were analysed in static images of the phantoms for each of the three fluoroscopic systems (Table 3). A significant difference in scores was observed between the different systems, with an average score of at least 3 out of 5 at a concentration Ta of 7.5% for the Artis Pheno, 12.5% for the Azurion and 20% for the Discovery.

**Table 3:**
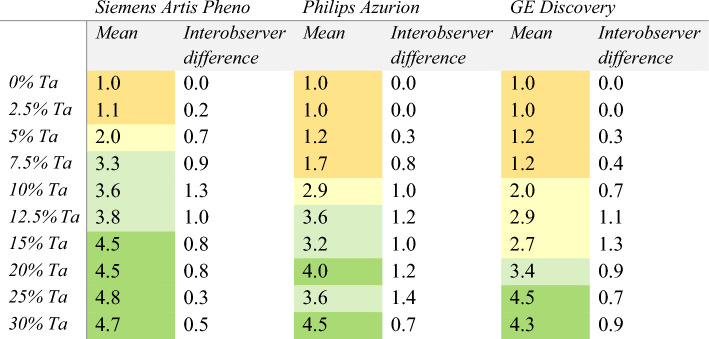
Average scores, from 1 to 5 (1 = very poor, 5 = excellent), and interobserver difference of the polymer detectability in the 2 mm channel, evaluated by clinical experts

The scores are color-coded (<2: orange, 2 – 2.9: yellow, 3 – 3.9: light green, ≥ 4: dark green).

The interobserver difference for the detectability scores is shown in Table 3. Again, the difference was generally higher for intermediate concentrations. The intra-observer difference was 0.3.

## Discussion

Data from the present study show that concentrations of ≥ 20% Ta were consistently detected within the first 5 mm after entering the side branch. 15% Ta was on average detected within the first 5 mm, but not consistently, which may render insufficient for low dose scan protocols. Lowering the concentration to 20% may thus improve the usability and diagnostic accuracy of follow-up CT, the gold standard for endoleak detection, by reducing beam hardening and artifacts, while retaining sufficient detectability of the polymer for safe injection during fluoroscopy. However, differences were found in detectability scores of fluoroscopy images on different fluoroscopic systems, which resulted from differences in hardware, scan protocols and image processing for each manufacturer. The optimal Ta concentration in AneuFix for sufficient detectability may therefore differ between hospitals, and the Ta concentration, scan protocol and postprocessing should be adjusted accordingly in clinical practice.

Other injectable embolic agents that use Ta for radiopacity include the well-established Onyx (Medtronic, Minneapolis (MN), USA) and Squid (Emboflu, Gland, Switzerland). While Onyx and Squid were developed for neurological interventions, they have also been used in abdominal applications, such as embolization of type I and type II endoleaks [12–15]. Both are precipitating techniques, which require long preparation times, while insufficient mixing may result in inhomogeneities in the radiopacity [16], generally requiring higher Ta concentrations to ensure sufficient detectability across the region of interest. Beam hardening and streak artifacts during follow-up with CT have been reported for both Onyx and Squid [17, 18].

For Onyx, a study about the optimization of the radiopacity has been published by Treitl et al. [19] Aortic endoleak phantoms, consisting of 3D printed tubes with a stent graft and various endoleaks, were placed in a thorax phantom with a simplified spine. Polymer was injected at a steady rate. The endoleak embolization was analysed by two radiologists, by indicating the moment they saw the first material at the catheter tip. They also rated the visibility of the polymer, similar to our study. Treitl et al. found that the Ta concentration could be lowered to 45-50% of its original concentration. While the original Ta concentration was not disclosed, Onyx has been known to use a concentration of 35% Ta [20]. Four radiologists evaluated beam hardening and visualization of reperfusion endoleaks. Concentration of < 50% of the original concentrations were found to have significantly improved CT results. The required concentration Ta for sufficient detectability found by Treitl et al. is comparable to the concentration found in the present study.

The impact of Ta concentration on postprocedural imaging will increase when larger volumes are needed, such as for sac embolization. Prophylactic sac embolization using coils and/or glue was found to reduce the incidence of T2EL, promote sac regression and resulted in less reinterventions, although T2ELs remained to occur [21–24]. Failure was associated with larger volumes and a lower concentration of coils, indicating that complete filling of the flow lumen outside the stent graft may be a driver for success [21]. Polymers that solidify in situ, as in the present study, may therefore be a good alternative to more conventional embolization methods, but in these circumstances an optimal (and lower) Ta concentration is even more important.

Several factors that may negatively influence the detectability in clinical practice were taken into consideration in this in vitro study. The endoleak phantom was placed inside a customized abdomen phantom for realistic attenuation and background during fluoroscopy. An important aspect was the inclusion of a clinically relevant 3D printed vertebral column, instead of a simplified one. The position of the endoleak phantoms was chosen such that the outflow channels were overlaying the vertebral column, which makes distinction between side branch and background more challenging. Furthermore, the default clinical lowest-dose imaging protocol was chosen. Protocols with higher image quality, and correspondingly higher radiation exposure – if warranted by a direct benefit to the procedure – may result in better detectability. With these design choices, the minimally required concentration Ta found in this study is for a worst-case scenario in terms of contrast, leaving room for improved contrast in clinical practice, and thus better detectability.

The endoleak phantoms were scanned with a Siemens Artis Pheno, which showed the best polymer detectability scores in the fluoroscopic system comparison, using the lowest-dose scan protocol for each system. Therefore, other systems in clinical practice may require a different scan protocol, higher radiation dose or higher Ta concentration for sufficient detectability. Total radiation exposure was not included in this study.

Lowering the concentration would improve the efficacy of follow-up imaging by reducing bream hardening artifacts, resulting from higher attenuation of low-energy photons in the continuous energy spectrum produced by standard CT. In addition to lowering the concentration Ta, modern CT techniques such as dual-energy CT (DE-CT) [25] and photon-counting CT (PC-CT) [26] could be considered for follow-up as well, to further reduce beam hardening artifacts by homogenizing the contribution of photons with different energies to the total signal. In DE-CT, the data can be decomposed and reconstructed as a virtual monochromatic image, in which all photons have the same energy and are therefore attenuated equally. On the other hand, photon-counting detectors are able to directly convert photon energy into an electrical signal. This allows equal weighting of photons with different energies, compared to traditional detectors that convert photon energy into light, weighting the photons proportional to their energy. These two techniques could help to significantly reduce beam-hardening artifacts, compared to standard CT.

## Limitations

The current study has several limitations. Several design choices were made for standardization and repeatability of the experiment. These factors may have positively influenced the detectability of the polymer, and thus present a limitation on the results of this study. The setup was placed inside a CT abdomen phantom representing a typical human abdomen, but not all aspects were taken into account. For example, the subcutaneous fat tissue was missing and obesity may result in higher attenuation and thus lower detectability. The phantom also did not include a stent graft. Finally, a continuous and constant polymer injection rate of 40 mL/min was used, whereas AneuFix uses a manual dispenser. While our continuous injection allows for standardization, polymer flow rates may vary in clinical practice.

The analysis was performed based on the assumption that the polymer should be detected within the first 5 mm. Clinically the length of the lumbar arteries varies per patient, and the penetration depth of the polymer also depends on the clinician’s reaction time after detection. The human detection time is typically 0.25 s and may vary per observer, which was not taken into account in this study.

Finally, lowering the radiopacity of the polymer decreases imaging artifacts, which was observed qualitatively from cone-beam CT scans on the endoleak phantoms (Supplementary Information S3). However, no quantitative analysis of imaging artifacts on standard CT was performed in this study. Artifacts and beam hardening are generally less on cone-beam CT than on standard CT.

## Conclusion

Detection within the initial 5 mm upon entry into the side branch was achieved consistently for concentrations of ≥ 20% Ta and the detectability in static images was scored sufficient (scores of at least 3 out of 5) for ≥ 15% Ta. The corresponding CNR values for these concentrations are 2.23 ± 0.21 and 1.98 ± 0.13, respectively. This suggests that the concentration of Ta in current AneuFix can be decreased to 20% while retaining satisfactory detectability for injection purposes. Because significant differences in fluoroscopic systems and scan protocols exist between hospitals, the combination of Ta concentration and scan protocol needs to be adjusted to the specific clinical setting for safe injection and optimal postprocedural follow-up.

## Supplementary Information

Below is the link to the electronic supplementary material.

Supplementary file1 (PDF 821 KB)

Supplementary file2 (MP4 3920 KB)

## Data Availability

The processed data in support of the results in this article are available within the article or supplemental materials. Unprocessed data and analysis scripts are available upon reasonable request to the corresponding author.

## References

[1] Antoniou, G. A., S. A. Antoniou, and F. Torella. Editor’s Choice–endovascular vs. open repair for abdominal aortic aneurysm: systematic review and meta-analysis of updated peri-operative and long term data of randomised controlled trials. *Eur. J. Vasc. Endovasc. Surg.* 59(3):385–397, 2020. 31899100 10.1016/j.ejvs.2019.11.030

[2] Dijkstra, M. L., et al. Incidence, natural course, and outcome of type II endoleaks in infrarenal endovascular aneurysm repair based on the ENGAGE registry data. *J. Vasc. Surg.* 71(3):780–789, 2020. 31443976 10.1016/j.jvs.2019.04.486

[3] van Rijswijk, R. E., et al. Predictors of abdominal aortic aneurysm shrinkage after endovascular repair. *J. Clin. Med.* 11(5):1394, 2022. 35268486 10.3390/jcm11051394 PMC8910935

[4] Antoniou, G. A., et al. Prognostic significance of aneurysm sac shrinkage after endovascular aneurysm repair. *J. Endovasc. Ther.* 27(5):857–868, 2020. 32589118 10.1177/1526602820937432

[5] Wanhainen, A., et al. Editor’s choice–European Society for Vascular Surgery (ESVS) 2019 clinical practice guidelines on the management of abdominal aorto-iliac artery aneurysms. *Eur. J. Vasc. Endovasc. Surg.* 57(1):8–93, 2019. 30528142 10.1016/j.ejvs.2018.09.020

[6] Ameli-Renani, S., V. Pavlidis, and R. A. Morgan. Secondary endoleak management following TEVAR and EVAR. *CardioVasc. Intervent. Radiol.* 43(12):1839–1854, 2020. 32778905 10.1007/s00270-020-02572-9 PMC7649162

[7] Ultee, K. H., et al. Editor’s choice–systematic review and meta-analysis of the outcome of treatment for type II endoleak following endovascular aneurysm repair. *Eur. J. Vasc. Endovasc. Surg.* 56(6):794–807, 2018. 30104089 10.1016/j.ejvs.2018.06.009

[8] Smorenburg, S. P., et al. Initial clinical experience with aneufix injectable biocompatible elastomer for translumbar embolization of Type 2 endoleaks. *J. Endovasc. Ther.* 2023. 10.1177/15266028231165731. 37073926 10.1177/15266028231165731 PMC11707960

[9] Dunsker, S. B., et al. Extraforaminal lumbar arterial anatomy. *Surg. Neurol.* 61(1):29–33, 2004. 14706372 10.1016/s0090-3019(03)00541-x

[10] Espahbodi, S., et al. Colour doppler ultrasound of the lumbar arteries: a novel application and reproducibility study in healthy subjects. *Ultrasound Med. Boil.* 32(2):171–182, 2006. 10.1016/j.ultrasmedbio.2005.11.006 16464662

[11] Harrell, F.E. and J.C. Slaughter, Chapter 16.1 Intra- and inter-observer disagreement, in biostatistics for biomedical research. 1987: E-book: https://hbiostat.org/bbr/obsvar.html.

[12] Chun, J.-Y., and R. Morgan. Transcatheter embolisation of type 1 endoleaks after endovascular aortic aneurysm repair with onyx: when no other treatment option is feasible. *Eur. J. Vasc. Endovasc. Surg.* 45(2):141–144, 2013. 23276679 10.1016/j.ejvs.2012.11.010

[13] Müller-Wille, R., et al. Transarterial embolization of type II endoleaks after EVAR: the role of ethylene vinyl alcohol copolymer (Onyx). *Cardiovasc. Intervent. Radiol.* 36:1288–1295, 2013. 23397186 10.1007/s00270-013-0567-5

[14] Martin, M. L., et al. Treatment of type II endoleaks with onyx. *J. Vasc. Intervent. Radiol.* 12(5):629–632, 2001. 10.1016/s1051-0443(07)61489-4 11340144

[15] Venturini, M., et al. Transcatheter embolization with squid, combined with other embolic agents or alone, in different abdominal diseases: a single-center experience in 30 patients. *CVIR Endovasc.* 2(1):1–13, 2019. 32026992 10.1186/s42155-019-0051-7 PMC6966379

[16] Jiang, Y. Y., et al. In vitro quantification of the radiopacity of onyx during embolization. *Neurointervention*. 12(1):3–10, 2017. 28316864 10.5469/neuroint.2017.12.1.3 PMC5355458

[17] Pop, R., et al. Beam hardening artifacts of liquid embolic agents: comparison between squid and onyx. *J. NeuroIntervent. Surg.* 11(7):706–709, 2019. 10.1136/neurintsurg-2018-014542 30567844

[18] Schmitt, N., et al. Imaging artifacts of liquid embolic agents on conventional CT in an experimental in vitro model. *Am. J. Neuroradiol.* 42(1):126–131, 2021. 33214178 10.3174/ajnr.A6867 PMC7814783

[19] Treitl, K. M., et al. Reduction of CT beam hardening artefacts of ethylene vinyl alcohol copolymer by variation of the tantalum content: evaluation in a standardized aortic endoleak phantom. *Eur. Radiol.* 25:597–605, 2015. 25319348 10.1007/s00330-014-3438-9

[20] Siekmann, R. Basics and principles in the application of Onyx LD liquid embolic system in the endovascular treatment of cerebral arteriovenous malformations. *Intervent. Neuroradiol.* 11:131–140, 2005. 10.1177/15910199050110S117 PMC3404755 20584468

[21] Piazza, M., et al. Outcomes of endovascular aneurysm repair with contemporary volume-dependent sac embolization in patients at risk for type II endoleak. *J. Vasc. Surg.* 63(1):32–38, 2016. 26432285 10.1016/j.jvs.2015.08.049

[22] Fabre, D., et al. Prospective, randomised two centre trial of endovascular repair of abdominal aortic aneurysm with or without sac embolisation. *Eur. J. Vasc. Endovasc. Surg.* 61(2):201–209, 2021. 33342658 10.1016/j.ejvs.2020.11.028

[23] Wu, Y., et al. Systematic review and network meta-analysis of pre-emptive embolization of the aneurysm sac side branches and aneurysm sac coil embolization to improve the outcomes of endovascular aneurysm repair. *Front. Cardiovasc. Med.* 9:947809, 2022. 35935638 10.3389/fcvm.2022.947809 PMC9354492

[24] Dosluoglu, H. H., et al. Pre-emptive nonselective perigraft aortic sac embolization with coils to prevent type II endoleak after endovascular aneurysm repair. *J Vasc. Surg.* 69(6):1736–1746, 2019. 30591300 10.1016/j.jvs.2018.10.054

[25] Yu, L., S. Leng, and C. H. McCollough. Dual-energy CT–based monochromatic imaging. *Am. J. Roentgenol.* 199:S9–S15, 2012. 23097173 10.2214/AJR.12.9121

[26] Willemink, M. J., et al. Photon-counting CT: technical principles and clinical prospects. *Radiology*. 289(2):293–312, 2018. 30179101 10.1148/radiol.2018172656

[27] Nagel, J., et al. Optimizing the radiopacity of an injectable polymer for treatment and prevention of type II endoleaks. *J. Vasc. Surg.* 77(4):10S, 2023.

