# Supplementary material for: Optimizing the Radiopacity of an Injectable Polymer on Fluoroscopy used for Treatment of Type II Endoleak After Endovascular Aneurysm Repair
Source: Cardiovasc Eng Technol. 2025 Mar 24;16(4):377–85. doi: 10.1007/s13239-025-00779-w (PMC12367875; doi:10.1007/s13239-025-00779-w)
Supplement: Supplementary file 1 — Supplementary file1 (PDF 821 KB) [file 13239_2025_779_MOESM1_ESM.pdf]

SUPPLEMENTARY INFORMATION

Manuscript title:

Optimizing the radiopacity of an injectable polymer on fluoroscopy used for treatment of type II endoleak after endovascular aneurysm repair

Journal:

Cardiovascular Engineering and Technology

Authors:

Jeffrey R. Nagel [1,2], Erik Groot Jebbink [1,3], Stefan P.M. Smorenburg [4], Arjan W.J. Hoksbergen [4], Rutger J. Lely [5], Michel Versluis [2], Michel M.P.J. Reijnen [1,3]

[1] Multi-Modality Medical Imaging group, University of Twente, Enschede, The Netherlands

[2] Physics of Fluids group, University of Twente, Enschede, The Netherlands

[3] Department of Surgery, Rijnstate, Arnhem, The Netherlands

[4] Department of Surgery, Amsterdam UMC location Vrije Universiteit, Amsterdam, The Netherlands

[5] Department of Radiology, Amsterdam UMC location Vrije Universiteit, Amsterdam, The Netherlands

Please address all correspondence to Erik Groot Jebbink, e.grootjebbink@utwente.nl.

S1. Protocol for evaluation by clinical experts

Fluoroscopy videos of the polymer injection into the endoleak phantoms and static fluoroscopy images of the multi-vendor measurements were evaluated by clinical experts from several hospitals in the Netherlands. Six interventional radiologists and three vascular surgeons participated in the evaluation, from the Rijnstate hospital (Arnhem, Netherlands), Amsterdam UMC (Amsterdam, Netherlands), MST hospital (Enschede, Netherlands) and ZGT hospital (Almelo, Netherlands).

The fluoroscopy videos and images were randomly and evenly divided over nine data sets (Tables S1 and S2), with each data set containing each Ta concentration at least once and also including duplicates

for intra- and interobserver variability assessment. For the videos of the polymer injection, the moment that the polymer entered either of the side branches was evaluated by pausing the video and recording the time. The ground truth time of the polymer entering the side branches, obtained from video recording of the injection procedure that was synchronized with the fluoroscopy measurement, was subtracted from the experts' times to obtain the delay between the polymer entering the side branch and it being detected. These results were plotted including the standard deviation.

Table S1: Division of the fluoroscopy video data over the evaluation sets for the clinical experts. Experts evaluated each concentration at least once, with duplicates for inter- and intra-observer variability analysis.

Ta %	*1 **	*2*	*3*	*4*	*5*	*6*	*7*	*8*	*9*	*10*
Blank										
0%		✓	✓	✓	✓	✓	✓	✓	✓	✓
Series 1										
2.5%				✓			✓✓	✓		✓
5%				✓	✓		✓	✓		
7.5%			✓	✓			✓	✓		
10%				✓✓			✓✓		✓	
12.5%				✓			✓		✓✓	
15%				✓		✓	✓		✓	✓
20%				✓✓			✓			✓
25%		✓		✓			✓			✓
30%				✓			✓			✓✓
Series 2										
2.5%		✓			✓		✓			✓
5%		✓✓			✓					✓
7.5%		✓			✓			✓		✓

10%		✓		✓	✓			✓		
12.5%		✓✓			✓✓			✓		
15%		✓			✓			✓✓		
20%		✓			✓	✓		✓	✓	
25%		✓			✓✓				✓	
30%		✓	✓		✓				✓✓	
Series 3										
2.5%			✓	✓		✓			✓	
5%		✓	✓			✓			✓	
7.5%			✓✓			✓			✓	
10%			✓			✓			✓	✓✓
12.5%			✓		✓	✓			✓	✓
15%			✓✓			✓✓				✓
20%			✓			✓	✓	✓		
25%			✓			✓		✓✓		
30%			✓			✓✓		✓		

Table S2: Division of the static fluoroscopy images over the evaluation sets for the clinical experts. Experts evaluated image at least once (grey check marks), with duplicates for inter- and intra-observer variability analysis (green check marks).

Ta %	*1 **	*2*	*3*	*4*	*5*	*6*	*7*	*8*	*9*	*10*
Siemens Artis Pheno										
0%		✓	✓	✓	✓	✓	✓	✓	✓	✓
2.5%		✓✓	✓	✓	✓	✓	✓	✓✓	✓	✓
5%		✓	✓	✓	✓	✓	✓✓	✓	✓	✓
7.5%		✓	✓✓	✓	✓	✓	✓	✓	✓	✓✓

10%		✓	✓	✓✓	✓	✓	✓	✓	✓	✓
12.5%		✓	✓	✓	✓✓	✓	✓	✓	✓	✓✓
15%		✓	✓	✓	✓	✓✓	✓	✓	✓	✓
20%		✓	✓✓	✓	✓	✓	✓	✓✓	✓	✓
25%		✓	✓	✓	✓	✓	✓✓	✓	✓✓	✓
30%		✓✓	✓	✓	✓✓	✓	✓	✓	✓	✓
Philips Azurion										
0%		✓	✓	✓	✓	✓	✓	✓	✓	✓
2.5%		✓	✓	✓	✓✓	✓	✓	✓	✓	✓
5%		✓	✓	✓	✓	✓	✓✓	✓	✓	✓✓
7.5%		✓✓	✓	✓	✓	✓	✓	✓	✓	✓
10%		✓	✓	✓✓	✓	✓	✓	✓	✓	✓✓
12.5%		✓	✓✓	✓	✓	✓✓	✓	✓	✓	✓
15%		✓	✓	✓	✓	✓	✓✓	✓	✓✓	✓
20%		✓✓	✓	✓	✓	✓	✓	✓	✓✓	✓
25%		✓	✓	✓	✓	✓✓	✓	✓	✓	✓
30%		✓	✓	✓✓	✓	✓	✓	✓✓	✓	✓
GE Discovery										
0%		✓	✓	✓	✓	✓	✓	✓	✓	✓
2.5%		✓	✓✓	✓	✓	✓	✓	✓	✓✓	✓
5%		✓	✓	✓	✓	✓✓	✓✓	✓	✓	✓
7.5%		✓	✓	✓	✓✓	✓	✓	✓	✓	✓
10%		✓	✓	✓✓	✓	✓	✓	✓	✓✓	✓
12.5%		✓	✓	✓	✓	✓✓	✓	✓	✓	✓
15%		✓	✓✓	✓	✓	✓	✓	✓✓	✓	✓
20%		✓	✓	✓	✓✓	✓	✓	✓	✓	✓
25%		✓✓	✓	✓	✓	✓	✓	✓	✓	✓✓

30%		✓	✓	✓✓	✓	✓	✓	✓✓	✓	✓

The detection of the polymer was categorized in three regions; within 5 mm of entering the side branch (Figure 2.7, green region), between 5 – 10 mm (Figure S2, yellow region) and after 10 mm (Figure 2.7, red region). Safe detection was defined as a detection within 5 mm after the polymer enters the side branch. Ta concentrations for which the polymer was detected in the green region were considered sufficiently detectable.

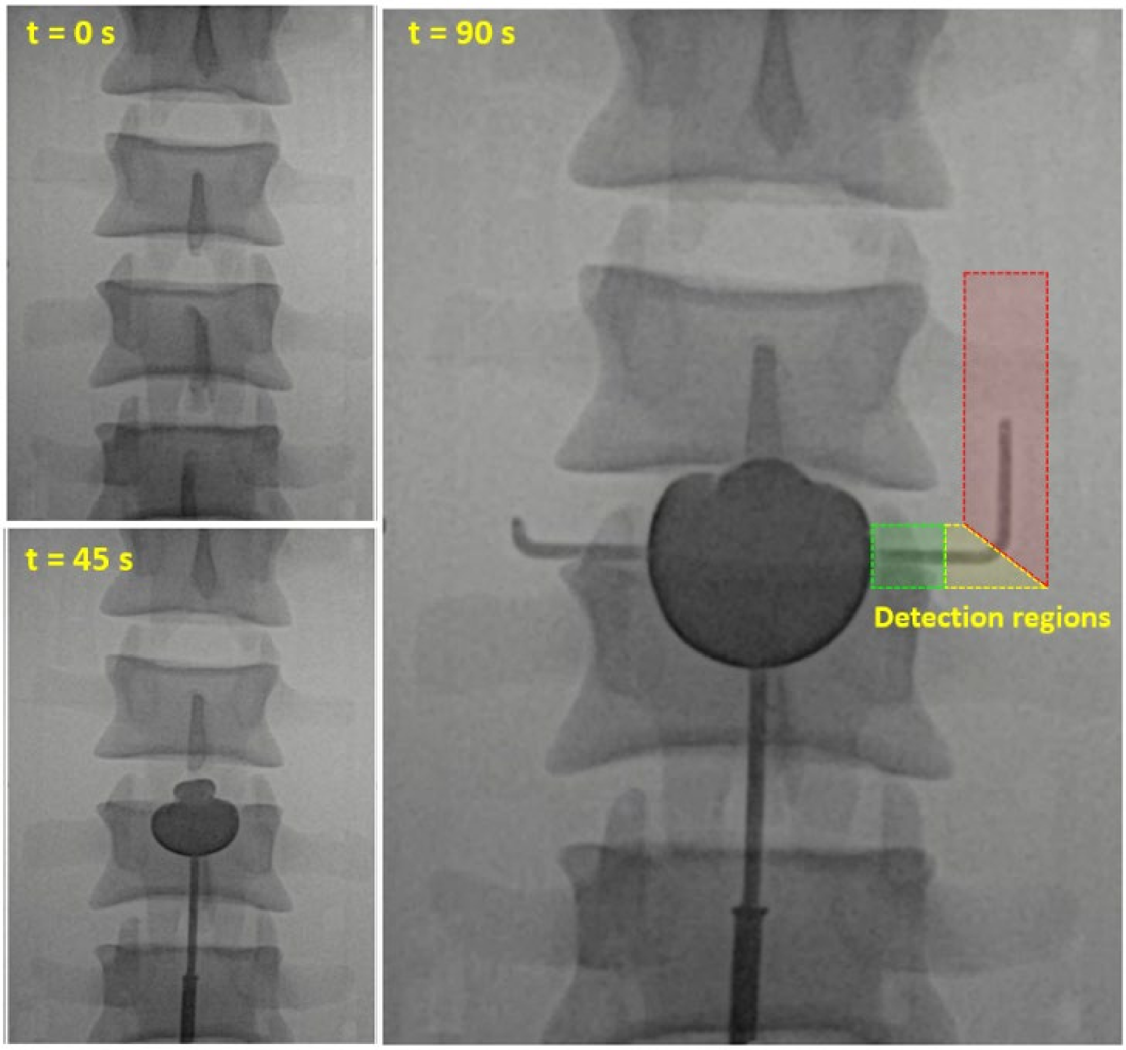

Figure S2: Polymer injection in the endoleak phantom at several time steps, with the final image showing the classification for the detection regions; green = within 5 mm of the channel origin, yellow = between 5 – 10 mm, red = after 10 mm.

The presence of the spine may obscure the side branches, both in the phantoms in this study and in clinical practice. Therefore, the clinical experts were also asked to score the polymer detectability at the side branch entry point and in the more peripheral region, without the spine in the background, on a scale of 1-5, with 1 being very poor detectability and 5 being excellent detectability (Figure S3).

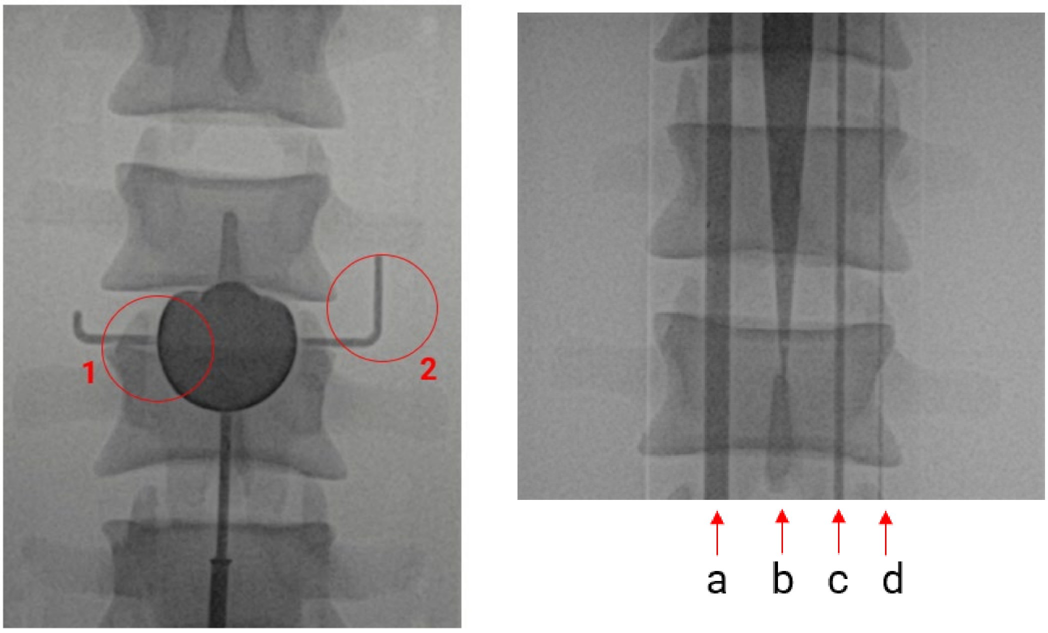

Figure S3: Left: regions in endoleak phantoms for detectability scoring; right: channels in multi-vendor phantoms for detectability scoring.

For the multi-vendor fluoroscopy images, 35 images were evaluated per expert, where each of the channels was scored, again from 1-5. Each expert evaluated all of the multivendor measurements (30 images) and duplicates (5 images) were included for intra-observer variability assessment.

Finally, the experts were shown a range of images from the polymer injections, ordered from low to high concentration. Experts were blinded for concentration and asked to indicate the threshold for sufficient detectability amongst these images.

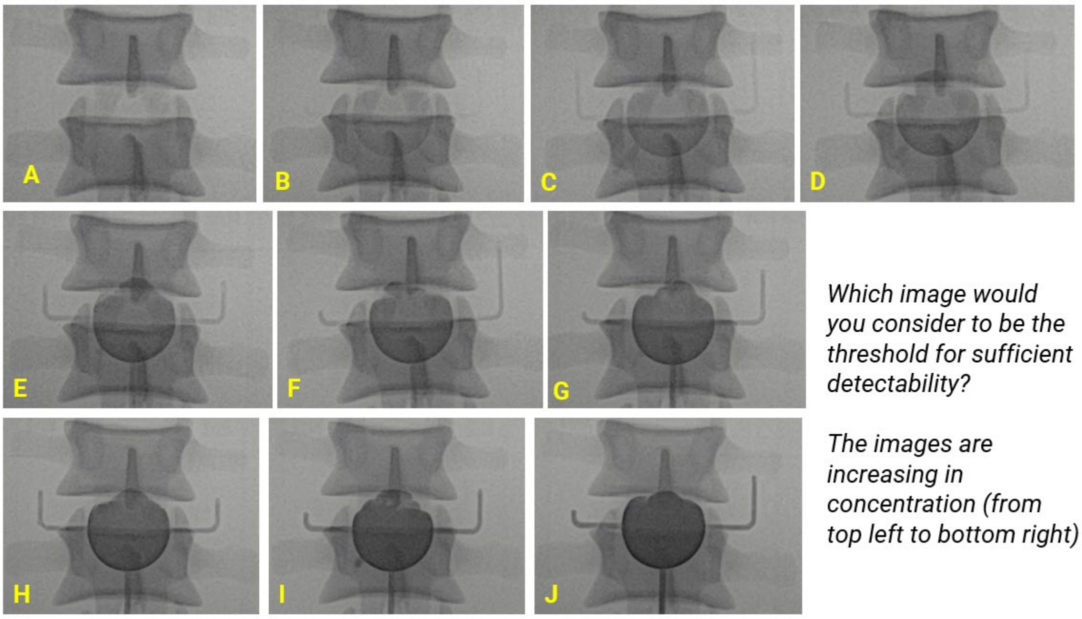

Figure S4: Final evaluation question, showing polymer injection images in increasing Ta concentration.

S2: Example fluoroscopy video of polymer injection

An example of a fluoroscopy video from the injection of polymer into the endoleak phantom has been attached in the supplemental files. This is the video for 30% Ta, measurement series 2, as evaluated by the clinical experts.

Only the part of the injection around the time that the polymer entered the side branch was evaluated (because with the full videos, the evaluation would have taken too long), with a random amount of time (3-10 seconds) between the start of the video and polymer entering the side branch.

Caption for Video S1:

Video S1: Fluoroscopy video of the injection of polymer with 30% Ta in the phantom, as shown to clinical experts for evaluation.

S3. Qualitative scatter reduction on cone-beam CT

To qualitatively investigate the scatter reduction as a result of lowering the Ta concentration, sac filling phantoms were fabricated consisting of a stent graft embedded in polymer (Figure S5a). The polymer cylinder includes three layers with different Ta concentrations – 10%, 12.5% and 30% – which are separated by layers of clear polymer for easy distinction between layers. A stent

graft (Endurant II from Medtronic, Fridley, USA) was placed inside the polymer and the cylinder was placed inside the abdomen phantom (Figure S5b) for a realistic attenuation and background on the CT scans.

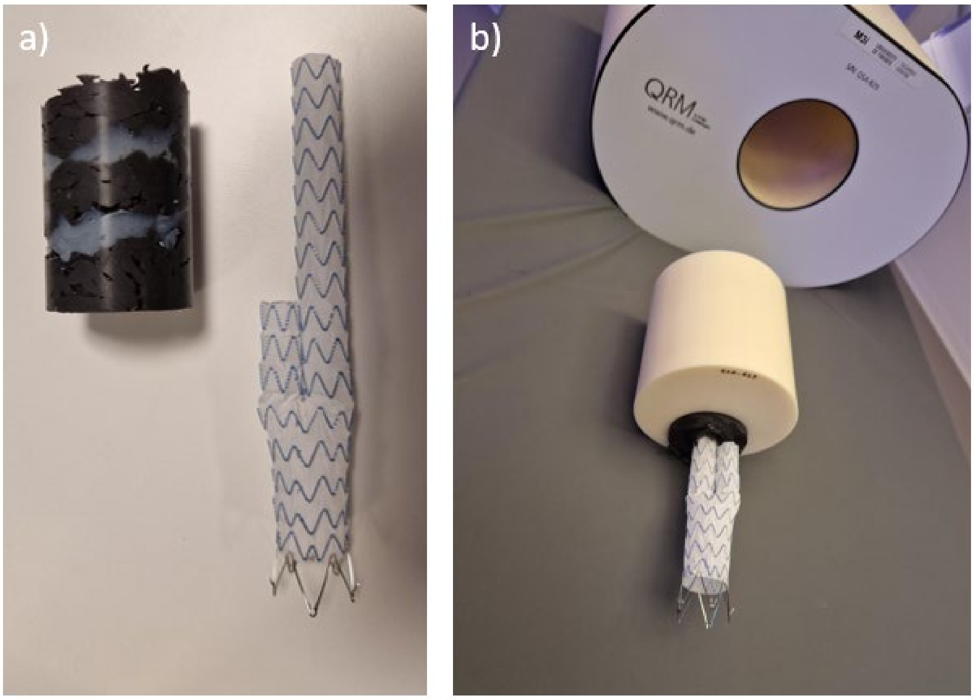

Figure S5: Sac filling phantoms, consisting of a stent graft embedded by a polymer cylinder (a), placed inside an abdomen phantom (b).

For the 30% Ta (Figure S6a), the stent graft is completely obscured and significant streak artifacts are visible. For 10% Ta (Figure S6b), the stent graft is visible and artifacts are reduced, but still present.

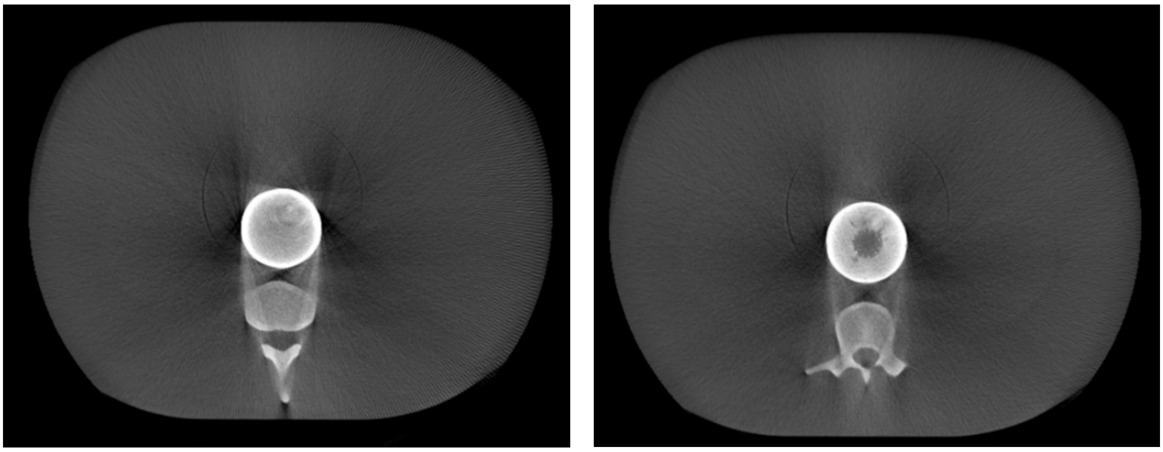

Figure S6: Cone beam CT of phantom with stent graft, for different concentrations Ta; 30% Ta on the left, 10% Ta on the right.
